# Case of Acute Opthalmitis, Etc.

**Published:** 1854-08

**Authors:** S. W. Thompson


### *Case of Acute Opthalmitis with Suppuration of the Globe of the Eye.*. By S. W. THOMPSON, M. D.

E. O. about the middle of February, 1854, came to me and requested my opinion upon an extensive opacity of the cornea of the right eye. He stated that some twelve or fourteen weeks previously, he was attacked with common sore eyes,—"Conjunctivitis."—That after the lapse of six weeks, the treatment he had undergone had done him no good, and the Cornea *then* ulcerated, and it was four weeks longer ere he could leave his room.

At the expiration of this period—"ten weeks in all"—the ulcerated surface healed, since which time his sight is entirely gone in the affected organ. He says there is no particular pain in the eye, but a sense of uneasiness and sometimes of weight. The cicatrix extended almost entirely across the cornea laterally, leaving a small portion of the superior surface apparently clear.

I told him I could give no encouragement as to the chance of its removal, nor even as to the partial recovery of his sight. I based my opinion, regarding the latter point, upon the probabilities of some of the internal structures of the eye being irreparably injured from the long continuance of the previous disease, and the still-existing sense of weight and uneasiness in the organ. I further told him, however, that it might be worth his while to pursue a proper and systematic course of treatment, to endeavor to remove the opacity, partially at least, before the cicatrix became hardened and beyond hope. He however left, without deciding to follow the advice. Drs. Whitlock and Turney saw the case at the same time, and concurred in the opinion I had expressed.

During the night of the 2d of March following, I was called to see him. He was suffering excruciating pain in the affected eye, and along the track of the supra orbital nerve of the same side. The whole eye-ball was greatly increased in size, protruding to a very considerable extent, and causing complete eversion of the inferior palpebra. Dr. Turney had been to see him twice previous to this date, and since this attack commenced, which was about three days ago. The bowels were costive, pulse weak but rather quick. Skin cool, but dry; urine high colored, and rather scanty; severe headache. The surface of the eye-ball was of a purplish red color. The superior palpebra was pushed up by the protruding organ, and presented a dark purplish, œdematous appearance. Profuse suppuration was going on from the whole exposed conjunctival surface. Cornea already sloughing. Dr. Turney had cupped him freely on the right temple, and had given mercurials to the extent of ptyalism.

I immediately ordered a hop poultice, and while this was being prepared, I made transverse scarifications upon the eye-ball, and then bathed the eye with warm water to encourage bleeding. Gave morphia $\frac{2}{3}$ gr., Dover's powder x. gr., to give him ease, and restore the cutaneous secretion. In the morning I found him easier, but the swelling of the eye-ball was not at all lessened. I ordered castor oil and turpentine, to evacuate the bowels; left tart. antim., to keep down vascular excitement, and morphia and Dover's powders, to quiet pain and nervous excitability; continue the warm water bathing, and the application of the hop poultice.

I was of the opinion that his eye would burst, as I believed there was matter contained within; I therefore advised him to submit to have it punctured through the already sloughing cornea, and thus evacuate the purulent accumulation. This, however, he objected to.

On the following day I saw him again. Condition not much changed. The surface of the eye-ball was lessened, but the everted eyelid was not reduced in thickness. Bowels open. Size of eye-ball not at all reduced. He could rest only whilst under the influence of the opiates. I still felt fearful the eye would burst, yet I could not enforce the measure, previously advised, to puncture, owing to the entire absence of all those symptoms which serve as landmarks to guide us in our diagnosis of the formation of matter. There had been no shiverings, no rigors, no night sweats, no sudden remission of pain and afterwards its recurrence. But I felt satisfied that during the attack he had previously suffered under, there had been a small accumulation of purulent fluid, which, when the eye was to all appearances free from disease, had been gradually increasing and causing the sense of weight and uneasiness.

I scarified the eye-ball again, as also the everted palpebra, and ordered a few drops of a solution of argenti nitras x. gr, aqua ℥j, to be introduced upon the schlerotic surface, and then to be immediately followed by bathing with warm water, as before. I used the argenti nitras in this form, because in several previous cases I had found it to answer better than anything else in reducing vascular engorgement. This plan of treatment, with such modifications as the case required, was continued for several days; I occasionally used the solid nitrate in place of the solution, wherever there was a disposition to unhealthy granulations upon the exposed conjunctival surface. He continued to improve in every respect, except the size of the eye-ball, which continued as great as before. I now, therefore, told the patient he must submit to have it punctured, or else there would ensue a still greater disorganization of the eye than had already taken place. The anterior laminæ of the cornea were entirely gone. There had been no symptoms during this attack, indicating the formation of pus, although at the latter part of the previous illness, he now tells me, he had shiverings, night sweats, &c., to a slight degree.

I accordingly introduced a narrow bistury through the remaining portion of the cornea, completely into the vitreous humor; this was followed by an escape of a sanguine purulent fluid. From that time he rapidly convalesced, and with much less sinking of the organ into the orbit than I anticipated, considering the length of time that elapsed for disorganization of the internal structures of the eye to take place, before the purulent secretion was evacuated.

I have given but an outline of the treatment, omitting the details other than such as I wished more particularly to mention. I might have used cups to the temples, but in their place I applied a blister behind the ear, and kept it open with stimulating dressings. I preferred this to cups because I considered the chief mischief was taking place in deeper seated textures than those to be reached by local depletion, and the derivative action of a blister could be kept up for a greater length of time than that of cupping. I am well aware that blisters in the acute stage of an inflammation are usually condemned by authorities; but as derivatives and counter irritants in opthalmia, I consider them invaluable, provided they are kept open until the inflammatory action is subdued; and in a case like this, where there exists no febrile action, they are doubly indicated. I prefer to apply them, in most cases, behind the ear, instead of the temple. You will remark here that I have used scarifications.

This, I am aware, is in opposition to the advice of very high authorities. Beer, Laurence, S. Cooper, and many others condemn them in the acute stage; but cases will sometimes occur in which we are constrained to fall back entirely upon our own judgment, even in violation of the general rules given us. So I considered it in this instance. Again, in regard to the use of the argenti nitras. I know this practice is condemned also in the acute stage; but, I am inclined to think, rather from theoretical than practical reasons; and I must confess that, so far as I may be allowed to judge from my own very limited experience, I know of no remedy which, under certain circumstances, at certain times, has such a powerful controlling effect over conjunctival inflammation as has this article, either in substance or solution. I do not limit its use to the sub-acute or chronic stages of the inflammatory process, nor to those cases alone in which there is mere redness or inflammation without much swelling or chemosis. The stage of inflammation alone does not govern me; it must be combined with other reasons for or against its use, ere I either use or discard it. Its application usually causes pretty severe pain at the moment; but this is soon relieved by warm bathing. The frequency of its repetition, as well as the strength of the solution, I judge of in each particular case.

